# Preparation and Evaluation of Modified Chitosan Nanoparticles Using Anionic Sodium Alginate Polymer for Treatment of Ocular Disease

**DOI:** 10.3390/pharmaceutics14122802

**Published:** 2022-12-14

**Authors:** Vaishnavi A. Bhosale, Vaibhavi Srivastava, Bhavana Valamla, Rati Yadav, Shashi Bala Singh, Neelesh Kumar Mehra

**Affiliations:** 1 Pharmaceutical Nanotechnology Research Laboratory, Department of Pharmaceutics, National Institute of Pharmaceutical Education and Research (NIPER), Hyderabad 500037, Telangana, India; 2 Department of Biological Science, National Institute of Pharmaceutical Education and Research (NIPER), Hyderabad 500037, Telangana, India

**Keywords:** fungal keratitis, chitosan, voriconazole, mucoadhesive, mucous penetrating, ophthalmic

## Abstract

Mucoadhesive nanoparticles offer prolonged drug residence time at the corneal epithelium by adhering to the mucous layer of the eye. Here, in this research investigation, voriconazole-loaded chitosan mucoadhesive nanoparticles (VCZ-MA-NPs) were modified to mucous-penetrating nanoparticles (VCZ-MP-NPs) by coating them with anionic polymer sodium alginate. The ionic gelation method was utilized to prepare mucoadhesive chitosan nanoparticles, which were further coated with sodium alginate to obtain the surface properties essential for mucous penetration. The developed VCZ-MA-NPs and VCZ-MP-NPs were evaluated extensively for physicochemical delineation, as well as in vitro and ex vivo studies. The particle size, polydispersity index, and ζ potential of the VCZ-MA-NPs were discovered to be 116 ± 2 nm, 0.23 ± 0.004, and +16.3 ± 0.9 mV, while the equivalent values for VCZ-MP-NPs were 185 ± 1 nm, 0.20 ± 0.01, and −24 ± 0.9 mV, respectively. The entrapment efficiency and drug loading were obtained as 88.06%±1.29% and 7.27% ± 0.95% for VCZ-MA-NPs and 91.31% ± 1.05% and 10.38% ± 0.87% for VCZ-MP-NPs, respectively. The formulations were found to be stable under different conditions (4 °C, 25 °C, and 40 °C). Chitosan nanoparticles and modified nanoparticles showed a spherical and smooth morphology under electron microscopic imaging. An excised caprine cornea was used for the ex vivo permeation study, exhibiting 58.98% ± 0.54% and 70.02% ± 0.61% drug permeation for VCZ-MA-NPs and VCZ-MP-NPs, respectively. The findings revealed that the mucous-penetrating nanoparticles could effectively pass through the corneal epithelium, thus overcoming the mucous barrier and fungal layer of the eye, which highlights their potential in the treatment of fungal keratitis.

## 1. Introduction

**Figure 1:**
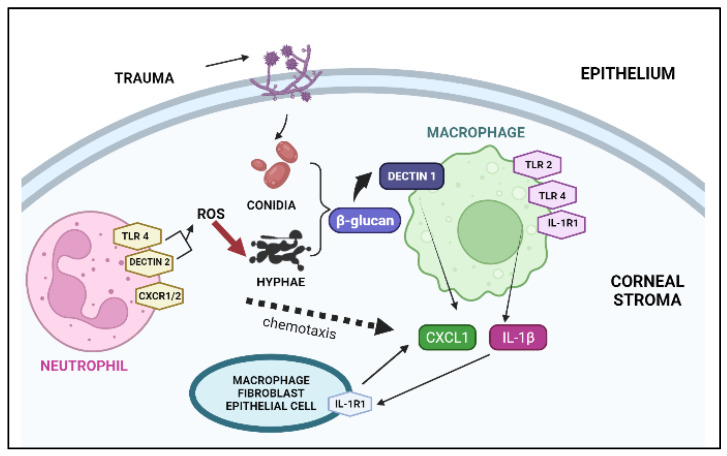
Corneal inflammatory sequences that occur in fungal keratitis. Injury to the corneal stroma encourages the inoculation of fungal conidia, which then germinate upon contact with β-glucan. Furthermore, β-glucan is detected by dectin-1 on macrophages that transcribe CXCL1 and IL-1β mRNA after a sequence of events. Upregulated CXC chemokines recruit neutrophils to the cornea, which stimulate ROS, resulting in fungal death. Abbreviations: TLR, Toll-like receptor; ROS, reactive oxygen species; IL, interleukin; CXCL-1, keratinocytes-derived cytokine.

Fungal keratitis (keratomycosis or mycotic keratitis) is a threatening corneal ailment, unquestionably brought on by filamentous fungi and yeasts [1,2]. According to recent studies and reports, there are one million instances of fungal keratitis per year, with Asia and Africa having the highest prevalence. Approximately 8–11% of affected people undergo eye enucleation, resulting in the removal of 84,000–115,000 eyes per year [3]. Filamentous fungi (*Fusarium*, *Aspergillus*, *Curvularia*, or other phaeohyphomycetes) and yeasts (*Candida albicans*) are the culprits behind fungal keratitis [4]. The occurrence of this disease is due to variable factors such as trauma, an immunocompromised state, and persistent usage of contact lenses [5]. Geographical (primarily occupational exposure) and etiological factors add to the variations in the pattern of the infection’s progression. Ocular surface irregularities and disorders, steroid use, prior ocular surgeries, and elementary diseases leading to an immunocompromised state are the leading factors underlying susceptibility to *Candida* spp. infections [6]. The use of contact lenses in temperate regions and exposure to industrial zones are among the major reasons for the growth of *Fusarium sp.*, which results in fungal keratitis [7]. Trauma predisposes the ocular surface to direct inoculation by fungi, which leads to infections in the corneal stroma causing fungal invasion [8]. Once fungi invade the tissue, they initiate replication in the stroma and penetrate deep inside the Descemet’s membrane, ultimately reaching the anterior chamber [4,9]. This fungal invasion causes maximal damage to the cornea, precipitating inflammation, hypopyon formation, and the formation of corneal scars that lead to opacity [10]. Corneal inflammation is a result of the emanation of inflammatory cytokines such as IL-17, IL-23, IL-8, IL-6, and IL-1β in human corneal epithelial cells (Figure 1) and neutrophils, in response to the adhesion of fungi to the corneal epithelium by surface proteins such as laminins, fibronectins, and collagens [11,12].

The topical use of natamycin and voriconazole represents the first-line therapeutic approach for fungal keratitis treatment. Like natamycin, amphotericin B also belongs to the polyene class of antifungal agents, making them both effective treatments for this ailment [13]. Nevertheless, these agents are also associated with acute toxicity, resulting in side-effects such as headache, vomiting, diarrhea, nausea, fever, and malaise that compromise patient compliance [14]. The echinocandin, an antifungal drug class is semisynthetic and contains cyclic lipopeptides with an N-linked acyl lipid side-chain, featuring high corneal penetration [13]. Caspofungin, micafungin, and anidulafungin are medications belonging to this class; however, they are only suitable for intravenous use as they exhibit poor oral bioavailability [15]. One of the most popular antifungal treatments for candida infection is 5-fluorocytosine; unlike other treatments, it is taken up by fungal cells and combined with their genetic material, consequently inhibiting the synthesis of DNA and RNA [13]. However, the therapeutic use of 5-fluorouracil (5-FU) is now restricted due to the rapid emergence of resistance [16].

Voriconazole is a second-generation, anti-fungal agent that belongs to the triazole class. *Aspergillus*, *Fusarium*, *Paecilomyces*, and other causative microorganism responsible for the development of fungal keratitis, they all are susceptible to voriconazole [17]. It acts by inhibiting the demethylation of lanosterol, that is a step in ergosterol synthesis in fungi, by binding to CYP51 (14-α- sterol demethylase) exhibiting fungistatic activity. It is thought to have increased affinity to 14-α-sterol demethylase, enhancing its activity in fluconazole-resistant organisms. Oral administration of voriconazole is complicated due to complex pharmacokinetics, noticeable drug interactions, and severe adverse effects; thus, topical drug delivery seems best treatment option for fungal keratitis [17,18].

Ophthalmic drug delivery is arduous under static and dynamic barriers such as reflex blinking, poor corneal permeability, dose spillage, epithelial drug transporters, tear secretion, and nasolacrimal drainage [19,20]. Efficient mucosal delivery remains challenging as mucus is an intimidating barrier to nanocarriers by entrapping and clearing them rapidly [21,22]. Mucoadhesive nanoparticles (MA-NPs) improve the pre-corneal dwell time and availability of the drug, which is beneficial over the conventional formulations that wear off due to tear drainage and dose spillage [23,24]. These perks of mucoadhesion are limited due to the shed-off rate or turnover time of the mucous layer of the eye, which is almost 5–10 min [25]. The MA-NPs become entrapped in the superficial coat of released mucin owing to their surface charge or size, which hinders their penetration deep through the mucous layer and further the underlying epithelium. It is responsible for less effective in treatment of diseases that demand deeper corneal drug penetration [26].

**Figure 2:**
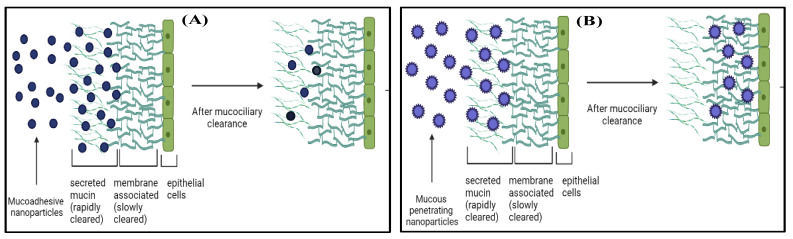
Graphical illustration of mucous layers and fate of (**A**) mucoadhesive, and (**B**) mucous penetrating nanoparticles after topical administration. MA-NPs adhere to the mucous layer of the cornea that clear off quickly with mucociliary clearance. At the same time MP-NPs penetrate deeper and tangle with static viscous mucous layer, thus lasting longer and proving more efficacious than MA-NPs.

In the case of fungal keratitis, the fungal invasion of epithelial cells of the cornea is underneath the mucous coating of the tear film. This mucous layer act as a barrier for the conventional formulations and MA-NPs to reach the infected corneal epithelium; thus, fungal growth continues to persist. The MP-NPs has tremendous potential to overcome the issue by piercing the mucous layer in the tear film and preventing the formation of fungus in the corneal stroma [10,26]. MP-NPs is a formulation approach to avoid mucoadhesion and steric obstructions, free from the entrapping in mucin and impale along-with the mucin layer that is comparably cleared slowly, thereby making the drug reach in the targeted underlying tissues [27,28,29]. MP-NPs should be appropriately sized to facilitate mucous pore diffusion and coated to avoid electrostatic interactions with the mucin fibers [30]. MP-NPs ensure more uniform drug delivery to the mucosal surfaces, reach the target tissues in higher concentrations (Figure 2), and sometimes extend the drug’s persistence time [21,26]. Various polymers that can be using for surface coating to achieve mucous penetration are Polyethylene glycol (PEG), Pluronic^®^ F-127 or Poloxamer^®^ 407, Polysarcosine (poly(N-methylglycine)), Poly(vinyl alcohol), Poly(2-alkyl-2-oxazoline), poly-(N-(2-hydroxypropyl) methacrylamide), Poly(2-hydroxyethylmethacrylate), and poly(2-hydroxyethylcrylate) [31,32,33]. Disruption of the mucous barrier using mucolytic agents is also an approach in designing of mucous penetrating nanoparticles. The mucolytics are used as an adjuvant to particle transport. Papain and bromelain are the tested mucolytics for mucous disruption.[34].

Chitosan is a linear polysaccharide comprising a deacetylated unit of β-(1→4)-linked D-glucosamine and an acetylated unit of N-acetyl-D-glucosamine, derived from chitin primarily present in *Didymella pinodes*, shells of shrimps, and other crustaceans by treatment with alkaline substances such as sodium hydroxide. In addition to providing a positive surface charge to nanoparticles, chitosan can also extend the contact time of therapeutics with the epithelium layer and enhances absorption through the para-cellular transport pathway.

Sodium alginate is a hydrophilic polysaccharide isolated from marine brown algae. It comprises (1–4) linked β-d-mannuronic acid (M units) and α-l-glucuronic acid (G units) exhibiting an anionic nature. It provides the mucous penetrating capability to nanoparticles by repulsive interactions with negatively charged sialic acid of the mucous layer [35].

The present research investigation’s main aim is to design and development of voriconazole-loaded MA-NPs and MP-NPs and evaluate for the fungal keratitis. Voriconazole-loaded chitosan nanoparticles were developed with mucoadhesive activity (VCZ-MA-NPs) and coated same with sodium alginate, giving mucous penetrating nanoparticles (VCZ-MP-NPs). These modified nanoparticles were evaluated for physicochemical characteristics such as pH, osmolarity, particle size, zeta potential, PDI, and spectroscopic techniques. In vitro drug release was performed using the dialysis membrane techniques. Ocular irritation potential was evaluated using Hen’s egg-chorioallantoic membranes (HET-CAM) assay. At the same time, an ex vivo corneal penetration study was carried out to compare the penetration ability of modified nanoparticles.

## 2. Materials and Methods

### 2.1. Materials

Voriconazole was accepted as a largesse sample from Aurobindo Pharma (Hyderabad, India). Chitosan (5–20 MPa·s viscosity grade) was obtained from TCI chemicals Ltd. (Mumbai, India). Sodium hydroxide and acetic acid was obtained from Avra Chemicals Ltd. (Hyderabad, India). Sodium tripolyphosphate (STPP) was procured from SRL Chemicals Pvt. Ltd. (Mumbai, India). Sodium alginate was procured from Loba Chemie Ltd. (Mumbai, India). Pluronic^®^ F-127 was purchased from Sigma Aldrich, Burlington, MA, USA. Sodium chloride, sodium bicarbonate, and calcium chloride dihydrate were procured from SRL Chemicals Pvt. Ltd. (Mumbai, India). Acetonitrile (HPLC grade) was purchased from Finar Ltd. (Ahmedabad, India). Triple distilled water was attained from Milli-Q^®^ (Evoqua, Pittsburgh, PA, USA).

### 2.2. Analytical Method Development by RP-HPLC

The shimadzu corporation (Kyoto, Japan) based RP-HPLC equipped with degasser (DGU-20A5R), SIL-20AC HT autosampler, column oven (CTO-10 AS VP), SPD-20A UV-VIS detector, and SPD-M40 PDA detector was used to develop the analytical method of voriconazole. C18 column of Shimadzu Shim-Pack Solar with dimension of 4.6 ID × 250 mm having a packed stationary phase of size 5µm was employed. Acetonitrile: water (pH adjusted to 4 by trifluoroacetic acid) in the ratio of 60:40 was employed as the mobile phase.

Solvent ratio as eluent was fixed precisely based on retention time (T_r_) and the tailing factor (T_f_) of the drug peaks. The flow rate 1 mL per minute was maintained, and the UV detector wavelength was adjusted at 256 nm. The acquisition time was kept to 10 min, and Lab solutions software was used for the method development. The stock solution of the drug was prepared in an acetonitrile, and the dilutions were spiked with an injection volume of 10 µL each. The method was validated as per International Conference on Harmonization (ICH) guidelines for linearity, precision, accuracy, limit of quantification (LOQ), and limit of detection (LOD). Triplicate dilution (*n* = 3) of each concentration was prepared and spiked in the column [36].

### 2.3. Preparation and Optimization of Blank (BC-NPs) and VCZ-Loaded Chitosan Mucoadhesive Nanoparticles (VCZ-MA-NPs)

**Figure 3:**
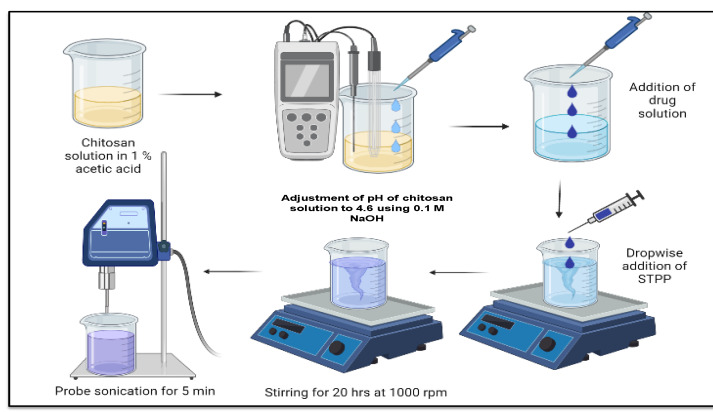
Schematic representation of ionic gelation method for the preparation of chitosan nanoparticles. Initially, chitosan solution was prepared and adjusted pH, followed by the addition of drug to it. Crosslinker was added dropwise and allowed to stir for overnight. Final formulation subjected to sonication for specific time.

The mucoadhesive nanoparticles were fabricated using ionic gelation method that involves cross linking of chitosan to STPP based on previously reported methods with minor modifications [37]. Chitosan (5–20 MPa·s viscosity grade) solution was composed by dispersing it in acetic acid (1% *v*/*v*) that required overnight stirring, and then the pH was balanced to 4.6 employing 0.1 M NaOH solution. The blank chitosan nanoparticles (BC-NPs) were prepared by adding STPP (0.2% *w*/*v*) dropwise into this cationic phase. The resultant opalescent solution was kept for 20 h with continuous stirring on a magnetic stirrer (IKAMAG^®^, IKA, Staufen, Germany) to obtain rigid nanoparticles. The ratio of chitosan to STPP was optimized according to the previously reported method [38]. The opalescent nanosuspension was then sonicated for 5 min (05s on, 05s off, 40% amplitude) to obtain nanoparticles with uniform size ranges within the specified limits (Figure 3).

For voriconazole-loaded chitosan nanoparticles (VCZ-MA-NPs), the ethanolic drug solution (20 mg/mL) was added to the chitosan solution containing 10% Pluronic^®^ F-127 as stabilizer before the addition of STPP. Three parameters were considered for optimization of nanoparticles: (i) The investigation was conducted on how the pH of the chitosan solution influences to the formation of nanoparticles. The effect of pH adjustment to 4.6 ± 0.05 and without pH adjustment was observed. (ii) The stirring time for preparing the chitosan solution was considered as input factor for optimization. The effect of different stirring time (1, 4, 7, and 20 h) was evaluated. (iii)Sonication time was optimized as it affects the particle size of nanoparticles. The opalescent nanoparticles suspension was subjected to sonication for the next 5 min to see the effect on the polydispersity index (PDI) and particle size.

### 2.4. Preparation and Optimization of VCZ Loaded Mucous Penetrating Nanoparticles (VCZ-MP-NPs)

VCZ-MP-NPs were prepared by coating the VCZ-MA-NPs with a negatively charged polymer. The nanosuspension of VCZ-MA-NPs was coated with sodium alginate solution. The coating was conducted by drop-wise addition of nanosuspension to the sodium alginate solution under continuous stirring at 500 rpm. The optimization parameters considered for the fabrication of mucous penetrating nanoparticles were as below: (i) The concentration of the sodium alginate solution-at different concentration (10 mg/mL, 5 mg/mL, and 2 mg/mL) of sodium alginate solution were used and investigated the effect on PDI, particle size, and ζ potential. (ii) The mass ratio of sodium alginate solution to nanosuspension of voriconazole loaded chitosan nanoparticles. The mass ratio of 1:1, 1:2, 1:3, 1:4, and 1:5 of sodium alginate solution to nanosuspension was considered as the input variable for optimization.

### 2.5. In Vitro Characterization of VCZ-MA-NPs and VCZ-MP-NPs

#### 2.5.1. Particle Size, Polydispersity Index (PDI) and ζ Potential Measurement

The particle size, PDI, and ζ potential of blank chitosan, VCZ-loaded mucoadhesive (VCZ-MA-NPs) and VCZ-loaded mucous penetrating nanoparticles (VCZ-MP-NPs) were evaluated by Zeta sizer Nano ZS (Malvern Instruments Ltd., Almelo, UK). At 25°C, a conventional four mW He-Ne laser operating at a wavelength of 633nm was used for the measurement. The amount of sample used for the analysis remained constant at 1 mL. The equipment has zeta sizer software for particle size, zeta potential, and PDI analysis. The data were collected and represented as mean ± SD (*n* = 3).

#### 2.5.2. Entrapment Efficiency and Drug Loading

The entrapment efficiency and loading capacity of the developed nanoparticles were estimated using the centrifugation method as reported earlier [39]. Nanosuspension (1 mL) of the VCZ-MA-NPs and VCZ-MP-NPs were ultracentrifuge (Thermo Fischer Scientific, Waltham, MA, USA) for 1 h at 50,000 rpm by maintaining the temperature of 4°C. Obtained clear supernatant was collected for estimating the free drug concentration by HPLC at 256 nm. For drug loading, the nanosuspension of VCZ-MA-NPs and VCZ-MP-NPs were lyophilized (Martin Christ, Osterode am Harz, Germany), employing 5% mannitol as cryoprotectant. The lyophilized powder was collected and weighed. The percentage entrapment efficiency, and drug loading capacity were determined and calculated using the below-mentioned formula.

$$\%\;\mathrm{Entrapment}\;\mathrm{efficiency}=\frac{\mathrm{Total}\;\mathrm{drug}-\;\mathrm{free}\;\mathrm{drug}}{\mathrm{Total}\;\mathrm{drug}}\times 100$$

$$\%\;\mathrm{Drug}\;\mathrm{loading}=\frac{\left(\mathrm{Total}\;\mathrm{drug}-\mathrm{free}\;\mathrm{drug}\right)}{\mathrm{Total}\;\mathrm{Weight}\;\mathrm{of}\;\mathrm{nanoparticles}}\times 100$$

#### 2.5.3. Compatibility Study

In order to identify potential interactions between the drug and corresponding excipients, the excipients-drug compatibility study was conducted using FTIR spectrum analysis. ATR-FTIR (Perkin Elmer, Waltham, MA, USA) was used for FTIR analysis of the drug, excipients, and physical mixture of excipients and drug. The samples of BC-NPs, VCZ-MA-NPs, and VCZ-MP-NPs were analyzed by the ATR-FTIR (Perkin Elmer, Waltham, MA, USA) by compressing the force gauge and scanning the sample from a spectral range of 4000–400 cm^−1^.

#### 2.5.4. Nuclear Magnetic Resonance (NMR)

The samples were subjected to NMR (Bruker AVANCE 500 MHz, Austin, TX, USA) analysis by loading 5 mg of lyophilized samples in deuterated DMSO as the solvent. The spectrum of the formulations was compared with the spectrum of voriconazole, chitosan, and sodium alginate [40].

#### 2.5.5. pH and Osmolarity Measurement

The pH of the ocular formulation is essential to avoid irritation and side reactions. VCZ-MA-NPs and VCZ-MP-NPs were assessed using a pH meter (Mettler Toledo, Polaris Pkwy, OH, USA) at 25 °C for pH determination. Each sample was measured in triplicates and reported as mean ± SD [41].

The osmolarity of the nanosuspension was analyzed by a freezing point depression-based osmometer (Gonotec OSMOMAT 3000, Logan, UT, USA). Milli-Q^®^ water and 0.9% *w*/*v* sodium chloride solution were employed as standard. About 50 µL of test samples were taken in the measuring vessel and the probe were pushed into the freezing chamber after attaching the vessel to the thermistor. The vessel was subjected to freezing, and the resultant osmolarity was measured and displayed digitally. The data were collected and represented as mean ±SD (*n* = 3).

#### 2.5.6. Electron Microscopy

##### Scanning Electron Microscopy (SEM)

Scanning electron microscopy (FEI, Hillsboro, OR, USA) was used to analyze the surface morphology of VCZ-MA-NPs and VCZ-MP-NPs. Images were recorded using a scanning electron microscope with an Everhart-Thornley detector under a vacuum. The samples were posed on carbon adhesive tape to hold the samples on the stub, followed by gold coating for three consecutive cycles. The images were recorded at a voltage of 10.00 kV [42].

##### Transmission Electron Microscopy (TEM)

The size and surface of the VCZ-MA-NPs and VCZ-MP-NPs were determined by subjecting them to TEM (JEM-2100, JEOL, Akishima, Japan) by dilution and mixing with uranyl acetate (1% *w*/*v*). This mixture was applied on a carbon-coated copper grid and allowed to air dry for a few minutes. Excess samples were removed using tissue paper. The surface of the nanoparticles was observed by adjusting the appropriate magnification [42].

#### 2.5.7. Physical Stability Study

Blank chitosan NPs, VCZ-MA-NPs, and VCZ-MP-NPs were stored in glass vials and retained at cold (4 °C), optimal (25 °C), and accelerated (40 °C) environments for one month. This investigation was conducted to look for signs of stability problems such as color change, phase separation, and particle precipitation.

#### 2.5.8. In Vitro Drug Release Study

The VCZ-MA-NPs and VCZ-MP-NPs underwent in vitro drug release tests employing modified dialysis diffusion methods using a dialysis bag having 12kDa MWCO (Sigma Aldrich, Saint Louis, MI, USA).Freshly prepared STF (simulated tear fluid, pH 7.4) comprising sodium chloride (0.67 g), sodium bicarbonate (0.22 g), potassium chloride (0.14 g), and calcium chloride dihydrate (0.008 g) was employed as the diffusion medium (1000 mL).The dialysis membrane was activated by previously soaking overnight in the diffusion medium. One mL of the formulation was taken into the dialysis membrane and hermetically tied on both ends using membrane closures. The dialysis membrane was suspended in a Schott DURAN bottle containing 50mL of diffusion medium at (37 ± 0.5 °C). This assembly was shaken at 100 rpm in an incubator (KS 3000, IKA, Landsberger, Germany). A one mL sample was withdrawn at predefined intervals and replenished with an equivalent amount of fresh pre-warmed STF (pH 7.4 maintained at 37 ± 0.5 °C). Aliquoted samples were analyzed by HPLC at 256 nm. The data were collected and represented as mean ± SD (*n* = 3) [43].

#### 2.5.9. Ocular Irritancy Test

The ocular irritancy capability was determined by Hen’s egg chorioallantoic membrane test (HET-CAM test). Three eggs in each group of positive control (0.1 M NaOH), negative control (0.9% *w*/*v* NaCl), and test substances (VCZ-MA-NPs and VCZ-MP-NPs) were employed. Eggs from Lohman Brown hens that were fertilized 8 to 10 days earlier and weighed ranged 35–40 g was chosen, retained at 38.30±0.20°C and 58.00 ± 2.0% relative humidity (RH) in a regulated controlled incubator and candled to discriminate the defective eggs. The eggshell was cracked on the side facing the air sac, and the white shell was delicately removed using forceps. About 0.3 mL of the test samples were applied to the CAM, ensuring that more than half of the area of the CAM was covered with it. The reactions of the CAM to the nanosuspension were observed for hemorrhage, vascular lysis and coagulation. The irritation score analysis (ISA) was conducted at three endpoints of 0.5, 2, and 5 min. The scoring was calculated using the following equation and carried out using the conventional scoring methodology as described in Table 1 [44].

$$\mathrm{IS}=\left[\frac{\left(301-\mathrm{Hsec}\right)}{300}\right]*5+\left[\frac{\left(301-\mathrm{Lsec}\right)}{300}\right]*7+\left[\frac{\left(301-\mathrm{Csec}\right)}{300}\right]*9\;$$

#### 2.5.10. Ex Vivo Corneal Permeation Study

The ex vivo corneal penetration or permeation investigation was executed on eradicated goat cornea acquired from the local slaughterhouse. The goat cornea was collected and stored in the cold saline medium (0.9% *w*/*v* sodium chloride solution at 4 °C) until it was used. The goat eyes were rinsed in cold brine to remove the sticking or extra tissue. The cornea was carefully extracted from the eyeball with 2–4 mm of sclera tissue surrounding it, and this excised cornea (surface area 1.13 cm^2^) was fixed between the acceptor and donor compartment of the Franz diffusion cell apparatus (Orchid Scientific Pvt. Ltd., Nashik, India) with the acceptor compartment filled with 25 mL of STF. About 1 mL of VCZ-MA-NPs and VCZ-MP-NPs was placed on the cornea through the donor compartment. This assembly was set at 37± 0.5 °C with 100 rpm. Aliquants of both formulations were collected at predefined time intervals and analyzed using HPLC at 256 nm. The permeation studies were performed in triplicates for both mucoadhesive (MA-NPs) and mucous penetrating nanoparticles (MP-NPs) and reported the data as mean± SD [41].

Permeability flux was calculated using the following formula:

$$\mathrm{Js}=\frac{Q_{A}}{A\cdot t}$$

where A is the area for permeation (cm^2^), Q_A_ is the amount of drug permeated through the cornea (mg), Js is the drug flux (mg/cm^2^/h), and t is the exposure duration (h). Additionally, permeability (P) was determined using the equation:

$$P=\frac{\mathrm{Js}}{C_{d}}$$

where C_d_ denotes to total drug amount loaded into the donor compartment (mg).

### 2.6. Statistical Analysis

All the experiments were carried out in three replicates to establish the mean and standard deviation (SD) for each experimental value and interpreted through Graph Pad Instat Software using one-way ANOVA with a statistical significance of *p* ≤ 0.05 (Version 9.3.1, Graph Pad Software, San Diego, CA, USA).

## 3. Results and Discussion

Mucus is a sticky, viscoelastic, gelatinous substance that covers the mucosal tissues exposed to the external environment, secreted by the mucous glands, forming a mesh of mucin fibers (glycoproteins) that entraps topically delivered particles. Mucus majorly consists of water (90–98%), along with 0.2–5% of mucin, salts (1%), and a minimal amount of proteins (0.5%) [45]. Therefore, particle size and surface charge of topically delivered substances are influential role in the effective delivery of drugs to the mucosal systems. In the present study, the mucoadhesive nanoparticles are formulated from chitosan, a polycationic biopolymer that has properties of interaction with the negatively charged mucin, leading to mucoadhesion. 

These mucoadhesive nanoparticles are surface modified with sodium alginate to escape the mucoadhesion, penetrate through the mucous layer and reach in the deeper corneal epithelium to cure deeper corneal lesions. As we know, sodium alginatehaving a negative surface charge, repels the interaction with mucin (negative charge), therefore enhances the penetration of modified nanoparticles. The in vitro drug release and ex vivo corneal infiltration trials determine the release behavior and the corneal permeation of the nanoparticles, respectively. To prevent ocular irritancy, which was assessed by HET-CAM assay, the pH and osmolarity of the nanosuspension must be appropriate. Method development of Voriconazole was conducted using RP-HPLC. The calibration curve was recorded at a wavelength of 256 nm using the mobile phase of acetonitrile and water (pH 4 adjusted by TFA) in the ratio of 60:40 and flow rate of 1 mL/min. The linear plot was observed for concentrations ranging from 0.4–24 µg/mL. The drug eluted at 5.5 min from the column with R^2^value of 0.9994. The analytical method was accurate and précised to detect 0.0157μg of the drug (LOD) and LOQ value of 0.0478μg. 

### Preparation and Optimization of Nanoparticles

The optimization parameters, including pH and stirring time in manufacturing, were considered for the composition of BC-NPs by the ionic gelation method. The effect of the pH of the chitosan solution in the nanoparticles formation was studied with adjusting pH and without pH adjustment of the cationic chitosan phase. There is a critical pH for forming chitosan nanoparticles using the ionic gelation method for forming unimodal size ranged nanoparticles.

The chemical structure of chitosan contains a hydroxyl (-OH) and amine (-NH_2_) groups, responsible for the chemical derivatization and solubility of molecules. Acidic pH helps in protonation of the-NH_2_ group, which alters the charge of the macromolecule. Positively charged chitosan molecules can strongly interact with negatively charged sialic acid of mucin, causing mucoadhesive behavior. Thus, low pH of chitosan solution is necessary for obtaining mucoadhesion [46,47].

According to the previous literature, the pH of the chitosan solution plays a favorable role in the fabrication of the nanoparticles. Less amine protonation occurs at a crucial pH range, i.e., 4.6–4.8, constricting the chitosan chain and promoting the formation of smaller particle size nanoparticles [48]. The pH of freshly prepared chitosan solution was 3.5, which was adjusted to 4.6 using 0.1 M NaOH solution. Chitosan NPs showed particle size and PDI of 221 ± 16and 0.47 ± 0.03, respectively (Table 2).

Similarly, the stirring time for stabilization of the chitosan nanoparticles was optimized. The stirring time points of 1, 4, 7, and 20 h stirring was considered as the input factor as it can speed up the process of STPP dispersion in chitosan solution, and the enhanced shear force helps to make the mono-dispersity better (Table 3). The higher the stirring time, the higher the stabilization and shear force. 

Another optimization parameter is sonication time. Sonication involves the introduction of ultrasonic sound waves into the formulation used to travel in a compression and rarefaction manner. These high-intensity ultrasonic waves produce tiny vacuum bubbles that mature along with the propagation of waves. After reaching saturation, the bubble collapses violently with high pressure generating boundless energy, resulting in the depletion in molecular interaction. So, overall, sonication helps in reducing particle size through agitation. The opalescent suspension of VCZ-MA-NPs and VCZ-MP-NPs was subjected to sonication for periods of 1, 2, 3, 4, and 5 min to understand the effect on particle size and polydispersity index (Table 4). It was observed that the best result was obtained with 5 min sonication.

The nanosuspension of mucoadhesive nanoparticles was modified by coating with sodium alginate to impart surface properties of mucous penetration. The VCZ-MA-NPs was added drop-wise into the sodium alginate aqueous solution under continuous stirring at 500 rpm. The optimization parameters were considered, the mass ratio of sodium alginate solution to VCZ-MA-NPs and the concentration of the sodium alginate solution (Table 5). It was observed that the particle size and PDI were decreased upon increasing the amount of MA-NPs with respect to sodium alginate. Polydisperse larger particles have been formed upon the interaction of an equal amount of chitosan and alginate (1:1). However, the particle size decreases with an increasing the concentration of VCZ-MA-NPs. This is mainly due to additional alginate resulting in multiple coats of alginate over MA-NPs. Finally, it may conclude that the increasing of the overall particle size is due to repulsive interactions. While additional MA-NPs will electrostatically interact with negatively charged alginate and reduce the overall particle size. The negative zeta potential of MP-NPs is due to alginate’s negative carboxyl group (-COOH). Further fixing the mass ratio of 1:5, different concentrations of sodium alginate solution were evaluated, and found best result with the concentration of 2 mg/mL (Table 6). It was noticed that with decreasing the concentration of alginate solution, particle size reduces because of bulkiness of the alginate molecule.

The average particle size of optimized VCZ-MA-NPs and VCZ-MP-NPs was 116 ± 2 and 185 ± 1, respectively. Both of the nanoparticles showed monodispersity with PDI 0.23 ± 0.004 for VCZ-MA-NPs and 0.20 ± 0.01 for VCZ-MP-NPs.

**Figure 4:**
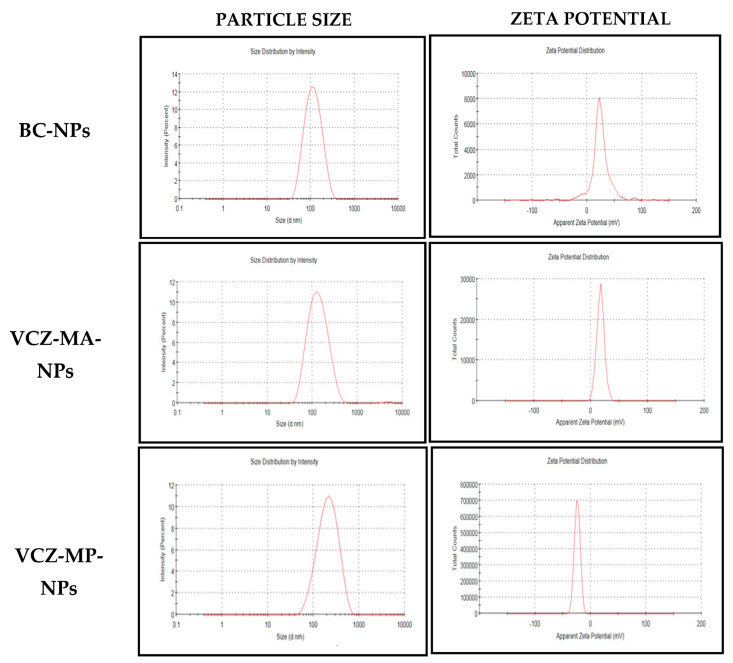
Graphs from dynamic lights catering (DLS) showing particle size, PDI, and zeta potential of optimized batches (BC-NPs, VCZ-MA-NPs, and VCZ-MP-NPs).

The zeta potential of VCZ-MA-NP showed a positive value of 16 ± 0.9 mV, while VCZ-MP-NP showed a negative potential of −24 ± 0.9 mV (Table 7). Negative zeta potential of the mucous penetrating nanoparticles can aid better penetration into the corneal epithelium through the mucous due to repulsive interaction with mucin glycoproteins (Figure 4).

**Figure 5:**
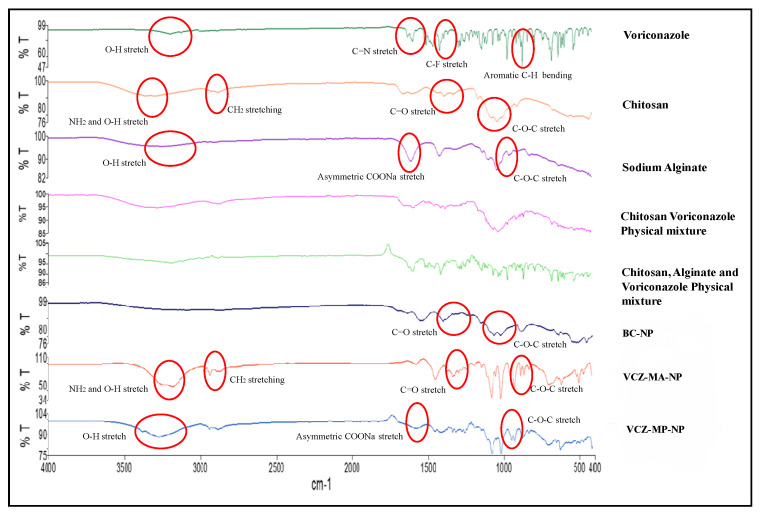
FT-IR spectra of voriconazole, chitosan, sodium alginate, physical mixture of drug and excipients, BC-NPs, VCZ-MA-NPs, and VCZ-MA-NPs (top to bottom). Characteristic peaks of VCZ disappeared in modified nanoparticles, while VCZ-MA-NPs, and VCZ-MP-NPs showed a similar pattern as chitosan and sodium alginate, respectively.

**Figure 6:**
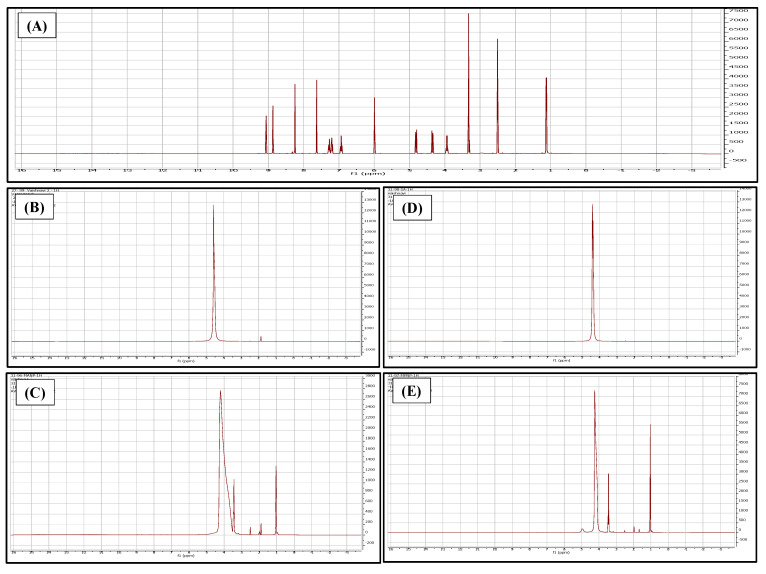
NMR spectra of (**A**) voriconazole, (**B**) chitosan, (**C**) VCZ-MA-NPs, (**D**) sodium alginate, and (**E**) VCZ-MP-NPs (where X axis is chemical shift in ppm while Y axis is intensity scale of spectral line).

The FT-IR spectrum of VCZ-MA-NP exhibits peaks at 3211, 2935, and 1082 cm^−1^ that are similar to those obtained in the spectrum of chitosan. There are no prominent peaks of voriconazole obtained in the spectrum of MA-NPs, indicating the encapsulation of the drug in the nanoparticles. The VCZ-MP-NPs spectrum exhibits peaks at 1589 and 958 cm^−1^, and the presence of no characteristic peaks of chitosan and voriconazole, ensuring the coating of the chitosan nanoparticles with sodium alginate. No extra peaks discovered in the physical mixture of drug and excipients confirm the compatibility of drug and formulation components (Figure 5). The NMR spectrum of VCZ showed that the hydroxyl group (OH) had a chemical shift of 5.98 ppm, and aromatic OH had a chemical shift of 7.28 ppm. The NH_2_group of the triazole ring had a chemical shift of 4.8 ppm. This confirms the purity of the drug. The NMR spectrum of VCZ-MA-NPs is similar to the chitosan NMR spectrum (δ 4.56 ppm) and drug’s peak. In VCZ-MP-NPs, the dominant peaks are 4.2, 3.45, and 1.04 ppm, the characteristics of sodium alginate and chitosan polymers. The absence of extra peaks confirms the lack of impurity (Figure 6). The optimum pH range reported for the ocular formulation is 6.6–7.8, and formulation outside this specified pH range can cause ocular irritation leading to lacrimation, pain, redness, and discomfort. The pH of the mucoadhesive and modified nanoparticles was found to be in the range of 6.9–7.1 and 7.0–7.2, respectively. Isotonicity of the ocular formulation is significant in preventing irritation and tissue damage. The osmolarity of VCZ-MA-NPs and VCZ-MP-NPs was determined to be in the range of 313 and 307 mOsmol/kg.

The percentage entrapment efficiency (%EE) of VCZ-MA-NPs and VCZ-MP-NPs was found 88.06 ± 1.29% and 91.31 ± 1.05%, respectively. However, the drug loading capacity of optimized VCZ-MP-NPs and VCZ-MA-NPs was found to be 10.38 ± 0.87% and 7.27 ± 0.95%, respectively. The mucoadhesive and mucous penetrating nanoparticles showed reasonable entrapment efficiency of voriconazole which is prominent for an effective drug delivery system. In determining the amount of drug present in one drop of optimized formulation, a single drop of the formulation was subjected to HPLC. VCZ-MA-NPs showed 0.14 ± 0.006mg/mL of drug concentration in its one drop (45–50µL), while one drop of VCZ-MP-NPs had 0.16 ± 0.003 mg/mL of voriconazole concentration.

**Figure 7:**
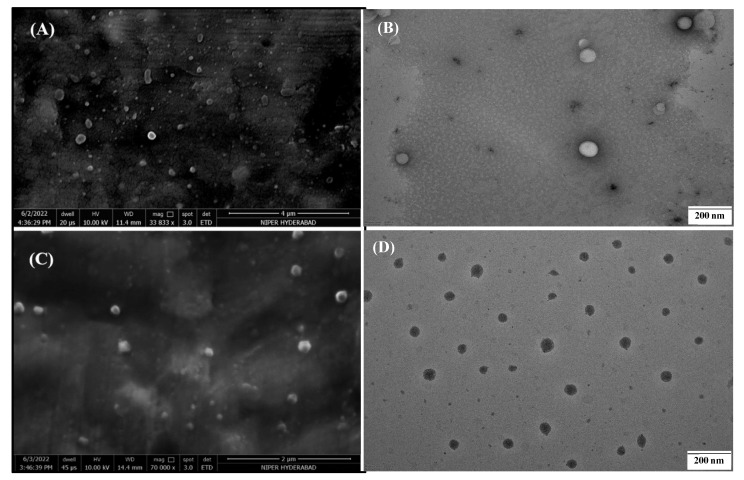
Photomicrographs of SEM (**A**)VCZ-MA-NPs, (**C**) VCZ-MP-NPs, and TEM Micrographs of (**B**) VCZ-MA-NPs, and (**D**) VCZ-MP-NPs.

The SEM micrographs (Figure 7A) of VCZ-MA-NPs and VCZ-MP-NPs showed spherical morphology and surface of the nanoparticles appears fine and smooth. The optimized formulations’ sizes were in same range determined by dynamic light scattering (115 nm in the case of VCZ-MA-NPs and 185 nm for VCZ-MP-NPs). TEM images of optimized VCZ-MA-NPs and VCZ-MP-NPs exhibited spherical surface shape and homogeneous particle size distribution. (Figure 7).

**Figure 8:**
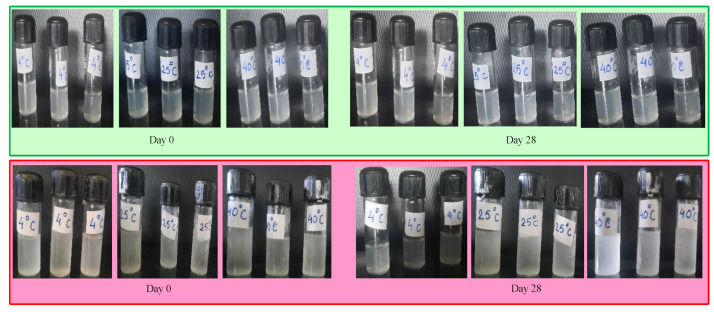
Physical stability of VCZ-MA-NPs (Green Box) and VCZ-MP-NPs (Pink Box) at three different temperatures (4 °C, 25 °C, and 40 °C). No significant changes were observed in terms of color change, turbidity, or drug precipitation during the one month stability study.

The VCZ-MA-NPs and VCZ-MP-NPs formulation were stored at three different temperatures and observed for one month in terms of alteration in color, odor, appearance, precipitation, etc. It was inferred that VCZ-MA-NPs and VCZ-MP-NPs both are physically stable with no sign of any alteration in physical appearance). No evidence of phase separation or precipitation was observed after an accelerated stability study of 28 days (Figure 8).

**Figure 9:**
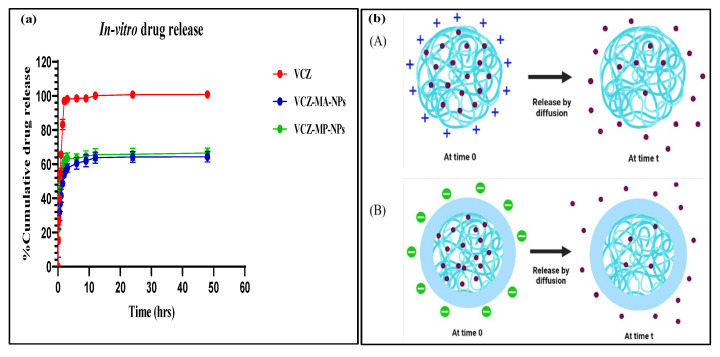
(**a**) In vitro percentage cumulative drug release from the VCZ solution, VCZ-MA-NPs, and VCZ-MP-NPs. Data are represented as mean ± SD (*n* = 3), (**b**) pictorial release mechanism from (A) VCZ-MA-NPs with positive surface charge, and (B) VCZ-MP-NPs having negative surface charge.

In vitro drug release was accomplished by dialysis bag technique under simulated condition of the eye using simulated tear fluid (STF) pH 7.4 comprising sodium chloride (0.67 g), sodium bicarbonate (0.2 g) and calcium chloride dihydrate (0.008 g) added to 1000 mL purified water and proper pH 7.4 adjustment. It was noticed that more than 80% of drugs discharged from the methanolic drug solution compared to the optimized formulations, i.e., VCZ-MA-NPs and VCZ-MP-NPs exhibited 48.79% and 57.50% of drug released, respectively, in 1.5 h. The modified nanoparticles exhibited controlled drug release for up to 48 h (Figure 9). The slow and sustained release is due to the diffusion of the drug from the chitosan core. There is 64.38±3.00% and 66.50 ±2.91% drug release from VCZ-MA-NPs and VCZ-MP-NPs after 48 h, while100%of drug released through methanolic drug solution within 12 h. To find the best-fitting kinetic model for VCZ-MA-NPs and VCZ-MP-NPs, the drug release statistics were exposed to various kinetic models, including first order, zero order, Korsmeyer–Peppas, and Higuchi. Both the formulation showed the same pattern of the kinetic model with the highest R^2^ value of the Korsmeyer–Peppas model; hence, it was the best-fit model. The order of regression coefficient (R^2^) for VCZ-MA-NPs and VCZ-MP-NPs is Korsmeyer–Peppas (0.6876 and 0.6032)> Higuchi model (0.5327 and 0.4260)> zero order kinetic (0.2686 and 0.1956)> first order kinetic (0.1035 and 0.0747). The release exponent obtained is 0.41 and 0.38 for VCZ-MA-NPs and VCZ-MP-NPs, respectively. As both are less than 0.45, that indicates Fickian diffusion is the governing element in the drug release process [49].

HET-CAM assay is an alternative test to the Draize rabbit eye test for determining the ocular irritancy potential of formulation using a chorioallantoic membrane (CAM) of the chick embryo. CAM is said to be the congenator of the human eye as it contains fine arteries and blood capillaries similar to human conjunctiva. HET-CAM assay is a well-entrenched model for determining the safety of pharmaceutical ophthalmic formulations. This might be because of the easy access and availability of hen’s egg compared to the mammalian eye model.

A fertilized hen’s embryo is secured by three protective layers: yolk sac membrane, amnion membrane, and chorioallantoic membrane. The yolk sac is in direct contact with the embryo providing nutrition and circulation of gases [50,51]. Amnion is a transparent membrane that maintains temperature and pressure of the embryo and protects the embryo from infection. The chorioallantoic membrane is a highly vascularized, extra-embryonic membrane exchanging gases and nutrients. In the HET-CAM assay, alteration in blood capillaries of CAM is observed and denoted as irritancy potential of test samples.

**Figure 10:**
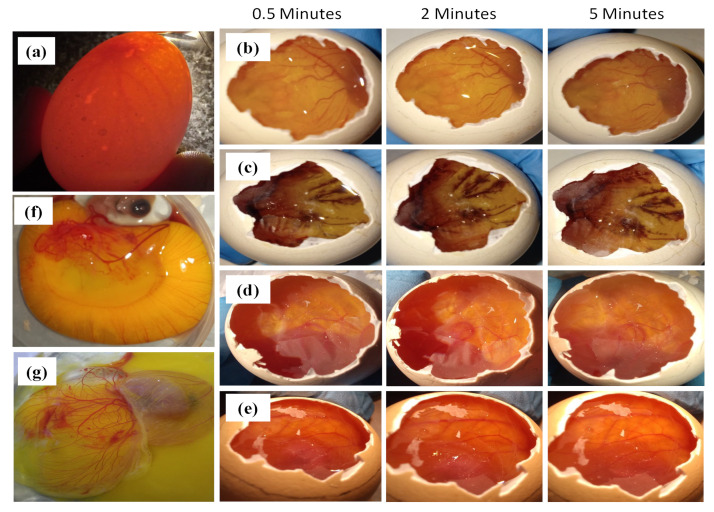
Pictures of HET-CAM Assay (**a**) candling of egg, CAM treated with (**b**) 0.9% *w*/*v* NaCl solution, (**c**) 0.1 N NaOH, (**d**) VCZ-MA-NPs, (**e**) VCZ-MP-NPs, and pictures of embryo after breaking internal membrane (**f**) VCZ-MA-NPs, (**g**) VCZ-MP-NPs.

The fertilized hen’s egg (8–10 days old) was grouped into four different classes. Each one of the groups was treated with 0.9% *w*/*v* NaCl solution as a negative control, 0.1 M NaOH (positive control), VCZ-MA-NPs, and VCZ-MP-NPs as test samples. It was observed that eggs treated with negative control had intact blood capillaries with no hemorrhage or lysis and showed an irritation score of 0. Positive control treated eggs showed hemorrhage just after 6 s with a very high irritancy score of 19. Treating CAM with VCZ-MA-NPs and VCZ-MP-NPs showed a similar picture as a negative control with an irritation score of 0 that confirms the safety of formulations for the eye (Figure 10). The irritancy score of both formulations was within the acceptable limit, and even after 5 min of exposure time, the capillaries were undamaged and flawless. The assay confirms that mucoadhesive NPs and modified NPs were safe to apply on the eyes. 

**Figure 11:**
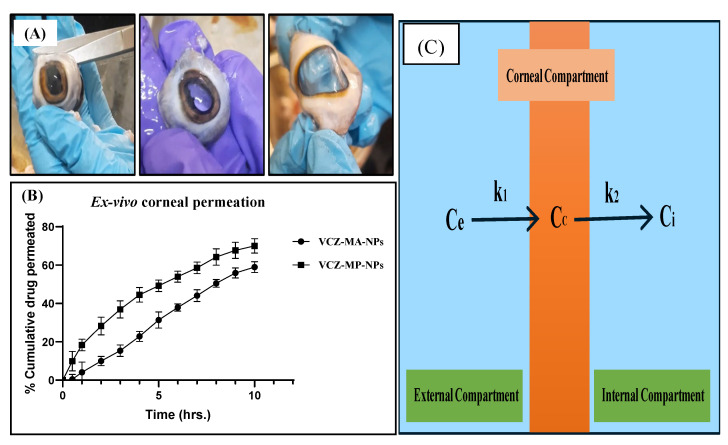
(**A**) Photographs for excision of goat cornea, (**B**) Ex vivo corneal permeation studies of VCZ-MA-NPs and VCZ-MP-NPs on excised goat cornea. Data are represented as mean ± SD (*n* = 3) and (**C**) Tricompartmental model of transcorneal permeation study. Ce is constant external or epithelial drug concentration, Cc: variable corneal concentration, Ci: variable internal or endothelial drug concentration, k1 and k2: rate constants from epithelial compartment to cornea and from cornea to endothelial compartment, respectively.

The ex vivo corneal permeation studies were performed for VCZ-MA-NPs and VCZ-MP-NPs on the excised goat cornea using Franz-diffusion cell apparatus. The percentage of cumulative drug permeated from VCZ-MA-NPs and VCZ-MP-NPs after 10 h is 58.98 ± 0.54% and 70.02 ± 0.61%, respectively (Figure 11B). The permeation flux of VCZ-MP-NPs is 1.18 times higher than VCZ-MA-NPs. The higher permeation and flux were achieved in the case of mucous penetrating nanoparticles. Drug permeated more efficiently through MP-NP than MA-NP due to coating of later with mucous penetrating polymer, i.e., negatively charged sodium alginate, as it enables the escape of nanocarriers from mucin interactions and easy penetration through the mucous layer of the tear film. The ex vivo corneal permeation data are in similar agreement with drug release studies performed in simulated tear fluid and suggesting that the VCZ-MP-NPs show better penetration and permeation capability than VCZ-MA-NPs ocular formulation. 

The drug permeation applies a tricompartmental model where a constant amount of drug-exposed to the cornea, but with time concentration of the drug gradually increases on the other side of the cornea, i.e., endothelial side or internal compartment (Figure 11C). As a result, the endothelium drug concentration has a time-dependent exponential slope. After a certain period, when steady drug flux begins, the tricompartmental framework with its five key components enters the scene. (i) C_e_ stands for external drug concentration;(ii) C_i_ stands for internal drug concentration.(iii) C_c_ stands for corneal drug concentration; (iv) rate constant from corneal to internal compartment k_2_; and (v) rate constant from external to corneal compartment k_1_(Depicted in Figure 11) [52]. The apparent transcorneal permeability can be calculated by following two kinetic equations:

$$\frac{{\mathrm{dC}}_{C}}{\mathrm{dt}}=k_{1}\left(C_{e}-C_{c}\right)-k_{2}\left(C_{c}-C_{i}\right)$$

$$\frac{{\mathrm{dC}}_{i}}{\mathrm{dt}}=k_{2\;}\left(C_{c}-C_{i}\right)$$

Simplifying the aforementioned equations by a sequence of mathematical operations, it was calculated and found that the apparent permeability of VCZ-MP-NPs through excised cornea was higher than the VCZ-MA-NPs, indicating the improved permeation through the mucous penetrating nanoparticles.

## 4. Conclusions

In the present work, chitosan and sodium alginate-based mucoadhesive and mucous penetrating nanoparticles loaded with voriconazole as an antifungal agent for the treatment of fungal keratitis were designed, developed, and exhaustively evaluated. The voriconazole-loaded chitosan mucoadhesive (VCZ-MA-NPs) nanoparticles were formulated by ionic gelation method, and the optimized formulation exhibited a mean particle size of 116 ± 2 nm, PDI of 0.23 ± 0.004 and ζ potential of + 16 ± 0.9 mV. These chitosan nanoparticles were modified as mucous penetrating by coating them with sodium alginate. The optimized mucous penetrating nanoparticles (VCZ-MP-NPs) show an average particle size of 185 ± 1 nm, PDI of 0.2 ± 0.01 and negative zeta potential of −24 ± 0.9 mV. The entrapment efficiency calculated for mucoadhesive and mucous penetrating nanoparticles is 88.06 ± 1.29%, and 91.31 ± 1.05%, respectively. Therefore, VCZ-MP-NPs contain more drug, as one drop of MP-NPs contains 0.16 ± 0.003 mg/mL drug concentration, while MA-NPs contain 0.14 ± 0.006 mg/mL of drug concentration. Microscopic images of optimized formulations showed spherical morphology with the same size as determined by DLS. The drug release from the VCZ-MA-NPs and VCZ-MP-NPs follows the Korsmeyer–Peppas kinetic model indicating the release mechanism of Fickian diffusion. The cumulative percentage of drug released from VCZ-MA-NPs was 64.38 ± 3.00%, while VCZ-MP-NPs showed a release of 66.50 ± 2.91% through 48 h in STF. Both formulations were found to be physically stable and safe for the eye in the HET-CAM assay. The percentage of cumulative drug permeated through excised goat cornea was 58.98 ± 0.54% and 70.02 ± 0.61% for mucoadhesive and mucous penetrating nanoparticles, respectively. The mucous penetrating nanoparticles showed better efficiency of entrapment, loading capacity, release kinetics, and permeation through the cornea than the mucoadhesive nanoparticles. Hence, it might effectively treat fungal keratitis and deep corneal ulcers as it can effectively cross the thick mucin layer owing to its modified particle size and surface potential. Based on the findings, VCZ-MP-NPs are proven to be potential nanocarriers for the treatment of fungal keratitis.

## Figures and Tables

**Table 1:** Scoring scheme for irritation testing with the HET-CAM test method.

Effect	Score		
	0.5 min	2 min	5 min
Hemorrhage	7	5	3
Vascular lysis	5	3	1
Coagulation	9	7	5

**Table 2:** Optimization of pH adjustment of chitosan solution for BC-NPs. Data are represented as mean ± SD (*n* = 3).

Batch	Particle Size (nm)	PDI
Without pH adjustment of the chitosan solution	559 ± 52	0.58 ± 0.02
**With pH adjustment to 4.6 using 0.1 M sodium hydroxide**	**221 ± 16**	**0.47 ± 0.03**

**Table 3:** Optimization of stirring time of chitosan solution for BC-NPs.

Batch	With pH Adjustment to 4.6	
Stirring Time	Particle Size ± SD (nm)	PDI ± SD
1 h	221 ± 16	0.47 ± 0.03
4 h	206 ± 16	0.45 ± 0.03
7 h	182 ± 02	0.41 ± 0.06
**20 h**	**155 ± 02**	**0.22 ± 0.01**

**Table 4:** Optimization of sonication time of chitosan solution for BC-NPs.

Batch	With pH Adjustment to 4.6 with 20 h Stirring	
Sonication Time	Mean Particle Size ± SD (nm)	Mean PDI ± SD
1 min	118 ± 1	0.27 ± 0.004
2 min	102 ± 1.7	0.20 ± 0.005
3 min	113 ± 0.8	0.28 ± 0.02
4 min	143 ± 4.1	0.35 ± 0.02
**5 min**	**99 ± 1.4**	**0.19 ± 0.002**

**Table 5:** Optimization of alginate to VCZ-MA-NPs mass ratio.

S. No.	Mass Ratio Alginate (10 mg/mL): VCZ-MA	Particle Size (nm)	PDI	Zeta Potential (mV)
1.	1:1	1583 ± 16	0.67 ± 0.15	−42 ± 2.1
2.	1:2	862 ± 17	0.68 ± 0.09	−40 ± 4.4
3.	1:3	758 ± 24	0.79 ± 0.21	−38 ± 5.0
4.	1:4	699 ± 22	0.95 ± 0.08	−33 ± 3.2
5.	**1:5**	**652 ± 11**	**0.55 ± 0.09**	**−34 ± 4.8**

**Table 6:** Optimization data of VCZ-MP-NPs.

Alginate Concentration	Mass Ratio Alginate: VCZ-MA-NPs	Particle Size (nm)	PDI	Zeta Potential (mV)
10 mg/mL	1:5	652 ± 11.2	0.55 ± 0.09	−34 ± 4.8
5 mg/mL	1:5	341 ± 13.1	0.43 ± 0.01	−29 ± 0.9
**2 mg/mL**	**1:5**	**185 ± 1**	**0.20 ± 0.01**	**−24 ± 0.9**

**Table 7:** Particle size, PDI, and zeta potential of optimized batches, (1) blank chitosan NPs, (2) VCZ-MA-NPs, and (3) VCZ-MP-NPs. Bold data are represented as mean ± SD (*n* = 3).

S. No.	Batch Specifications	Size (nm)	PDI	Zeta Potential (mV)
1.	Chitosan (5–20 Mpa) in 1 % acetic acid at 4.6 pH adjusted with 0.1 N NaOH and STPP (2 mg/mL), 5 min sonication	99 ± 1	0.19 ± 0.002	+15 ± 2.9
2.	Chitosan (5–20 Mpa) in 1 % Acetic acid at 4.6 pH adjusted with 0.1 N NaOH, VCZ (20 mg) and STPP (2 mg/mL), 5 min sonication	116 ± 2	0.23 ± 0.004	+16 ± 0.9
3.	Optimized VCZ-MA-NPs and sodium alginate solution (2 mg/mL) in 5:1	185 ± 1	0.20 ± 0.01	−24 ± 0.9

## Data Availability

Not applicable.
